# Evaluation of the Telomere Length in Patients with Spinal Muscular Atrophy

**DOI:** 10.3390/ijms262211223

**Published:** 2025-11-20

**Authors:** Betül Okur Altındaş, Sedat Öktem, Kürşat Bora Çarman, Mahmut Selman Yıldırım

**Affiliations:** 1 Department of Medical Genetics, Necmettin Erbakan University Medical Faculty Hospital, Konya 42090, Türkiye; drmselman@gmail.com; 2 Department of Medical Genetics, Dr. Ali Kemal Belviranlı Obstetrics and Gynecology Hospital, Konya 42285, Türkiye; 3 Division of Pediatric Pulmonary Diseases, Department of Pediatrics, Medipol Mega University Hospital, İstanbul 34214, Türkiye; soktem@medipol.edu.tr; 4 Division of Pediatric Neurology, Department of Pediatrics, Osmangazi University Medical Faculty Hospital, Eskişehir 26040, Türkiye; kbora@ogu.edu.tr

**Keywords:** spinal muscular atrophy, SMA, *SMN1*, telomere length

## Abstract

Spinal muscular atrophy (SMA) is an autosomal recessive neuromuscular disorder caused by biallelic *SMN1* gene loss, leading to motor neuron degeneration and progressive muscle weakness. The SMN protein is also implicated in telomerase biogenesis, suggesting a possible link between SMA and telomere regulation. This study aimed to investigate relative telomere length in pediatric SMA patients and evaluate, for the first time, the potential effects of gene replacement therapy with onasemnogene abeparvovec. Relative telomere length was measured in peripheral blood lymphocytes using quantitative real-time PCR in 58 patients and 58 age- and sex-matched healthy controls. Of the patients, 19 had received gene replacement therapy. SMA patients without this treatment exhibited significantly shorter telomeres compared with controls (*p* = 0.029), whereas no significant difference was observed between gene-treated patients and controls (*p* = 0.108). Direct comparison revealed longer telomeres in treated patients than in untreated ones (*p* = 0.012). These findings indicate that telomere attrition is present in SMA and may be mitigated by gene replacement. While the exact contribution of telomere biology to SMA pathogenesis remains to be clarified, telomere length may represent a biomarker for disease severity and treatment response, as well as a potential therapeutic target in this disorder.

## 1. Introduction

Spinal muscular atrophy (SMA) is an autosomal recessive neuromuscular disorder characterized by the progressive degeneration of alpha motor neurons in the anterior horn of the spinal cord. The disease arises due to deficiency of the survival motor neuron (SMN) protein, which is encoded by the *SMN1* gene [1]. A nearly identical gene, *SMN2*, acts as a disease modifier: because of a single-nucleotide difference affecting exon 7 splicing, *SMN2* primarily produces truncated, unstable SMN protein, with only a small fraction being full-length and functional. The number of *SMN2* copies largely determines disease severity [2]. Accordingly, SMA is clinically classified into types 0–4, ranging from the most severe prenatal or neonatal form (type 0) to the mild adult-onset form (type 4), depending on age at onset and maximum motor function achieved [1]. SMA represents one of the most common genetic causes of infant mortality and is associated with significant morbidity and mortality in pediatric populations [3]. Recent therapeutic advances have transformed the landscape of SMA treatment. Three disease-modifying therapies—nusinersen, risdiplam, and onasemnogene abeparvovec—have been approved by the United States Food and Drug Administration, offering renewed hope for affected individuals. These therapies target the underlying genetic defect through distinct molecular mechanisms. Nusinersen, an antisense oligonucleotide administered intrathecally, modifies *SMN2* pre-mRNA splicing by promoting the inclusion of exon 7, thereby increasing the production of functional SMN protein in the central nervous system [4]. Risdiplam, an orally administered small molecule, also enhances exon 7 inclusion in the *SMN2* transcripts and exerts systemic effects, resulting in elevated SMN protein expression levels both in the central nervous system and peripheral tissues [5]. In contrast, onasemnogene abeparvovec delivers a functional copy of the *SMN1* gene using an adeno-associated viral vector (AAV) 9, enabling sustained SMN protein expression primarily in motor neurons [6]. Although clinical outcomes have significantly improved [7], variability in treatment response and long-term molecular consequences of therapy remain subjects of ongoing investigation, highlighting the need for further studies into the broader cellular effects of SMN deficiency and its correction.

Telomeres are nucleoprotein complexes located at the ends of linear chromosomes, playing a crucial role in maintaining genomic stability. In mammals, telomeres consist of highly conserved hexameric TTAGGG DNA repeats [8]. Due to the unidirectional nature of DNA polymerase, replication of the lagging strand requires multiple RNA primers, resulting in the progressive loss of telomeric sequences with each cell division. Telomeres protect chromosome ends from being recognized as DNA damage and prevent the degradation of subtelomeric regions during replication. Once telomeres shorten to a critical length—approximately 4 kilobases—they trigger a DNA damage response that halts further cell division, contributing to cellular senescence. Telomere attrition has been implicated in aging, cancer, a wide spectrum of chronic diseases, and premature aging syndromes, and is considered both a hallmark and a potential driver of age-related decline in tissue function [9].

Telomerase, a specialized ribonucleoprotein complex, counteracts telomere shortening by adding TTAGGG repeats to the 3′ ends of chromosomes [10]. Telomerase activity is typically low or absent in most somatic cells but remains high in mitotically active cells such as stem cells, germline cells, and the majority of cancer cells, where it contributes to sustained proliferative capacity [11]. The biogenesis and assembly of telomerase take place in Cajal bodies—nuclear organelles known to serve as maturation centers for both telomerase and small nuclear ribonucleoprotein (snRNP) complexes [12]. The SMN protein, which is essential for snRNP assembly, is also critically involved in Cajal body function and has been shown to influence telomerase formation by modulating interactions between telomerase RNA and proteins such as dyskerin and coilin [13]. Experimental evidence has demonstrated that SMN directly interacts with telomerase components, suggesting that a role in telomerase regulation [14]. Despite these mechanistic insights, the link between SMA and telomere length remains largely unexplored. This study hypothesizes that telomere length may be altered in individuals with SMA due to the loss of SMN protein critical in telomerase biogenesis and Cajal body function, ultimately disrupting telomere homeostasis at the cellular level, contributing to cell cycle arrest and senescence. We further propose that gene replacement therapy, by restoring SMN protein levels, could mitigate such telomeric changes. Investigating these associations may provide insight into the molecular consequences of SMN deficiency and inform future research on the broader genomic effects of SMN-targeted therapies.

## 2. Results

### 2.1. Clinical and Demographic Characteristics of the Study Cohort

A total of 58 SMA patients and 58 healthy controls were included in the study. At the time of sample collection, the overall age distribution of the study cohort (n = 116) ranged from 7.5 to 212.5 months. In the patient group (n = 58), ages ranged from 7.5 to 208.3 months, and ages of the controls (n = 58) ranged from 8 to 212.5 months. No significant difference in age distribution was observed between patients and controls (U = 1642, Z = −0.221, *p* = 0.825).

**Figure 1:**
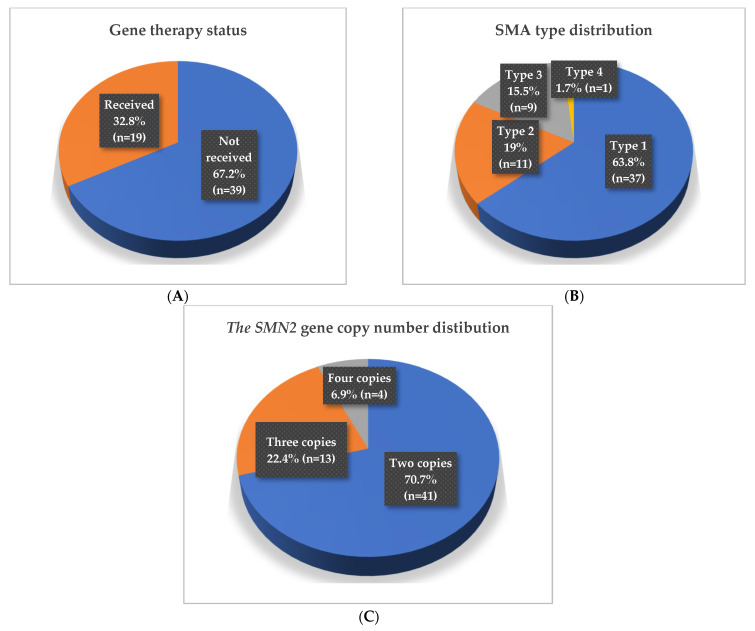
Patient cohort’s (**A**) gene replacement therapy status, (**B**) distribution of SMA types, and (**C**) distribution of the *SMN2* gene copy number.

Among patients, 19 individuals (32.8%) had received gene replacement therapy with Onasemnogene abeparvovec-xioi (ZOLGENSMA) (Figure 1A), with ages spanning 15.3–74.7 months. The remaining 39 patients (67.2%) had not received gene replacement, aged 7.5–208.3 months.

The patient cohort (n = 58) was further characterized in terms of SMA clinical types and the *SMN2* gene copy number. The majority of patients were diagnosed with SMA type 1 (n = 37, 63.8%), with a median age of 34.2 months; 18 of these patients (48.6%) had received gene replacement therapy. SMA type 2 was identified in 11 patients (19%, mean age: 75.3 months), of whom only one—a 30-month-old male—had undergone gene therapy. Patients with SMA type 3 accounted for 9 individuals (15.5%, mean age: 113.7 months), and a single patient (1.7%), a 20-month-old female, was diagnosed with SMA type 4 (Figure 1B).

Regarding the *SMN2* gene copy number, 41 patients (70.7%) carried two copies (median age: 38.6 months), including the 18 gene therapy recipients. Thirteen patients (22.4%) carried three copies (median age: 65.6 months), and only one had received gene therapy, while four patients (6.9%) carried four copies (mean age: 146.5 months) (Figure 1C).

Mean ages of the study participants according to gene replacement therapy status, clinical SMA type, and the *SMN2* gene copy numbers are summarized in Table 1.

The study population comprised 62 females (53.4%) and 54 males (46.6%). Patient and control groups were matched for sex distribution, with each group including 31 females (53.4%) and 27 males (46.6%), and no significant difference was observed between groups (χ^2^(1) = 0.000, *p* = 1.000). Among gene replacement therapy recipients, 11 (57.9%) were female and 8 (42.1%) were male, whereas in the untreated group, 20 (51.3%) were female and 19 (48.7%) were male. Considering SMA clinical subtypes, females accounted for 19 (47.5%) of type 1, 6 (54.5%) of type 2, and 5 (60%) of type 3 cases. Analysis of the *SMN2* copy number showed a comparable sex distribution: 56.1% of patients with two copies were female, 46.2% of those with three copies were female, and 50% of those with four copies were female.

### 2.2. Telomere Length Evaluation in Patients and Controls

Relative telomere length was analyzed to compare patients (n = 58) with their matched controls. The fold change values of all study participants, representing the log_2_-transformed relative telomere length obtained from qPCR using the formula 2^−ΔΔCt^ are presented in the Supplementary Table (Table S1). No significant difference was observed between the groups (Mann–Whitney *U* test, *p* = 0.346).

#### 2.2.1. Comparison by Gene Replacement Therapy Status

**Figure 2:**
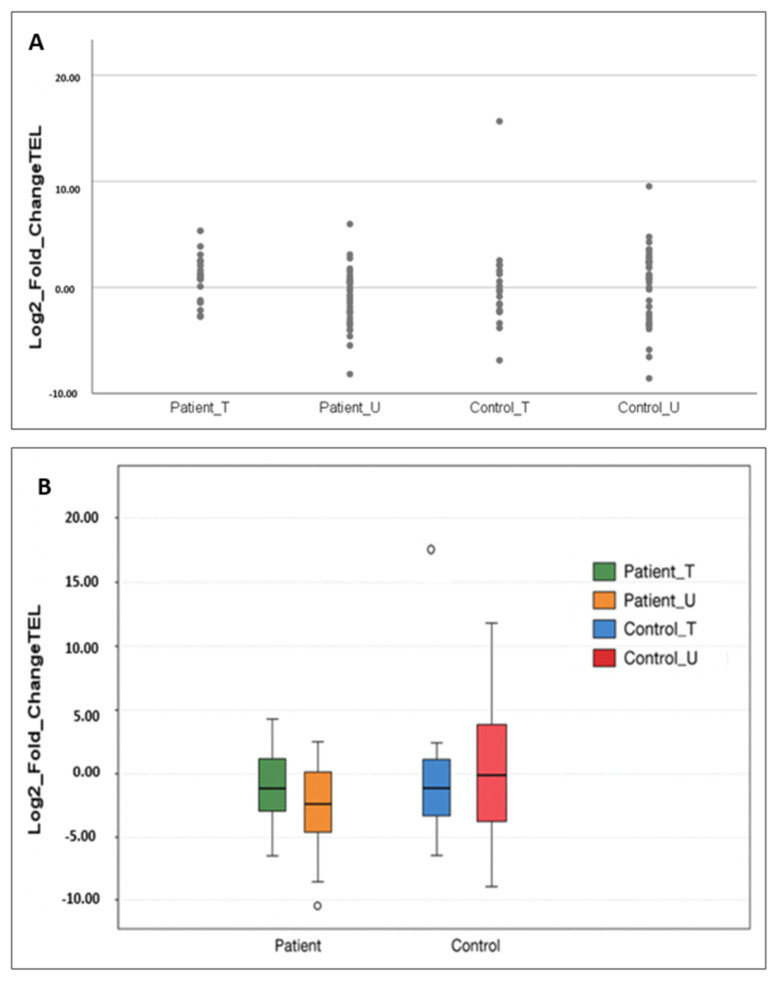
Patient_T refers to SMA patients who received gene replacement therapy, and Patient_U refers to those who did not. Control_T and Control_U indicate healthy individuals matched by age and sex to the treated and untreated patient subgroups, respectively. (**A**) Dot plot showing the log_2_ fold change in relative telomere length (calculated by the 2^−ΔΔCt^ method) for patients grouped by gene therapy status and their matched controls. Each dot represents an individual sample. (**B**) Box plot showing the log_2_ fold change in relative telomere length for patients grouped by gene therapy status and their matched controls. Boxes represent the interquartile range and the horizontal line indicates the median.

Patients were stratified into gene replacement therapy recipients (n = 19) and the other patients (n = 39). SMA patients who had not received gene replacement exhibited significantly shorter telomeres compared to their matched controls (*p* = 0.029), whereas gene therapy-treated patients showed no significant difference from controls (*p* = 0.108). Direct comparison between treated and untreated patients demonstrated a significant difference in telomere length (*p* = 0.012) (Figure 2, Table 2).

#### 2.2.2. Comparison by SMA Type

Telomere length did not differ significantly across SMA subtypes overall (*p* = 0.089). Excluding the single SMA type 4 patient also did not yield significance (*p* = 0.056). Among patients without gene replacement therapy, telomere length was not associated with SMA subtype (*p* = 0.471; *p* = 0.345 excluding the type 4 patient).

Within the cohort of gene therapy recipients, only SMA type 1 patients (n = 18) could be analyzed, with a mean fold change in telomere length of 1.09 ± 2.07; comparative analysis across other subtypes was not possible due to limited numbers.

When analyzed against matched controls, SMA type 1 patients (n = 37) showed no difference (*p* = 0.846). Untreated SMA type 1 patients (n = 19) did not differ from controls (*p* = 0.140), and treated type 1 patients (n = 18) also showed similar telomere lengths compared with controls (*p* = 0.090). A significant difference was observed when comparing treated vs. untreated SMA type 1 patients (*p* = 0.032).

SMA type 2 patients (n = 11) had shorter telomeres compared with controls (*p* = 0.004). This effect was driven by untreated type 2 patients (n = 10, *p* = 0.002).

SMA type 3 (n = 9) patients did not differ from their matched controls (*p* = 0.757) (Table 3). Spearman’s correlation analysis suggested a moderate negative correlation between telomere length and SMA severity (r = −0.334), although this was not statistically significant when stratified by treatment.

#### 2.2.3. Comparison by the *SMN2* Gene Copy Number

Telomere length did not significantly differ across *SMN2* copy number groups overall (*p* = 0.185) or among patients who did not receive gene replacement therapy (*p* = 0.864). In patients carrying two *SMN2* gene copies (n = 41), those who were untreated with gene therapy (n = 23) had significantly shorter telomeres compared with their controls (*p* = 0.024), whereas treated ones (n = 18) did not differ (*p* = 0.900). Comparison between treated and untreated two-copy patients also showed a significant difference (*p* = 0.010). No significant differences were observed in patients carrying three (n = 13) or four (n = 4) copies, regardless of therapy status (Table 4). Correlation analysis indicated no association between the *SMN2* copy number and telomere length.

#### 2.2.4. Correlation of Telomere Length with Age

**Figure 3:**
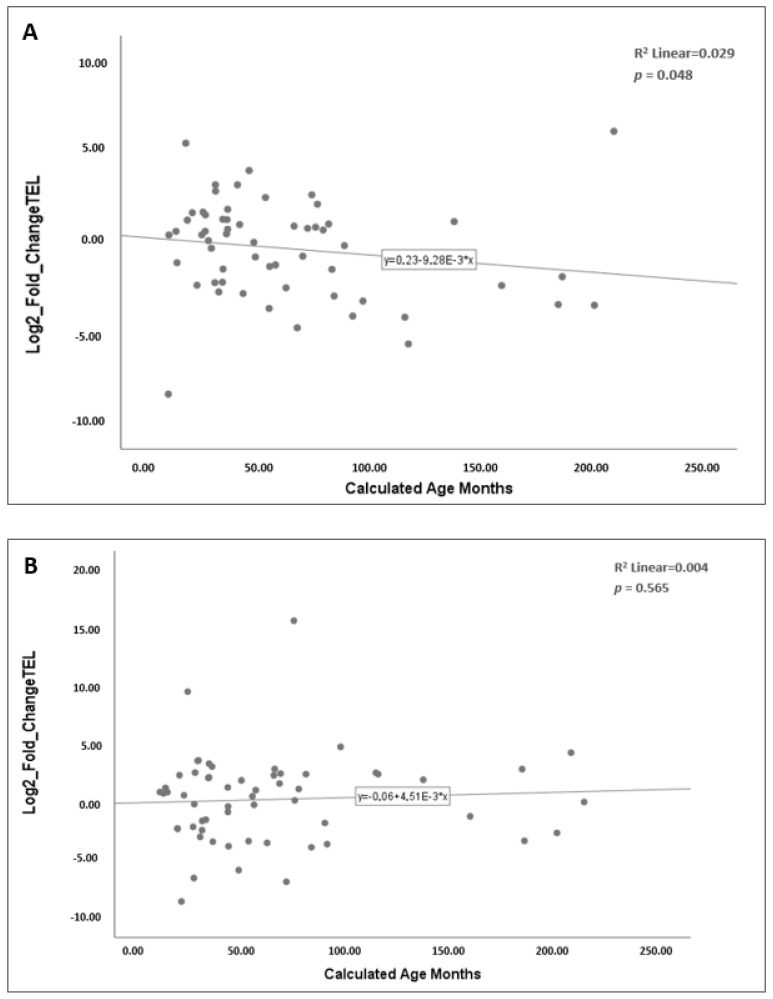
Correlation between relative telomere length and age in the study groups. The relative telomere length was determined using the 2^−ΔΔCt^ method and expressed as log_2_ fold change values on the y-axis. Each dot represents an individual sample, and the line indicates the linear regression fit. (**A**) Correlation in the patient cohort. (**B**) Correlation in the control group.

The relationship between age and telomere length was assessed using Spearman’s correlation. In the overall patient cohort, telomere length showed a weak negative correlation with age (*p* = 0.048, r = −0.26; Figure 3A), whereas no correlation was observed in controls (*p* = 0.565; Figure 3B).

When stratified by gene replacement therapy status, neither untreated (*p* = 0.486) nor treated patients (*p* = 0.870) exhibited significant correlations between telomere length and age.

## 3. Discussion

Telomeres serve two primary functions: protecting chromosome ends and preventing the loss of genetic material during DNA replication. Despite the existence of various mechanisms for telomere length maintenance, approximately 30–100 base pairs of telomeric DNA are lost with each cell division. In this context, once telomeres that function as buffer zones preserving the integrity of genetic information fall below a critical threshold, cells enter replicative senescence, ultimately triggering apoptosis [15]. Over the past decade, numerous studies have demonstrated associations between telomere length and various complex disorders [9]. However, the correlation between telomere length and spinal muscular atrophy (SMA)—a disorder characterized by motor neuron degeneration due to SMN protein deficiency—has not yet been sufficiently addressed in the literature. To our knowledge, the present study is only the second to evaluate telomere length in children with SMA and the first to examine the potential impact of gene replacement therapy on telomere dynamics.

The only study we were able to access that investigated the impact of telomere length in SMA patients was conducted in the Kashmir region of northern India, and a total of 98 individuals were included, consisting of 40 SMA patients and 58 healthy controls [16]. The mean age of patients (33.76 months) was significantly different than the controls (98.28 months). In terms of gender distribution, 52.5% of the patients and 38% of the controls were female. Relative telomere length in peripheral blood lymphocytes obtained from the participants was measured using the Monochrome Multiplex Quantitative Polymerase Chain Reaction method. In our study, the control group was selected after completion of patient recruitment to ensure complete age and gender matching, and relative telomere length was measured using the Real-Time PCR (qPCR) method. These two methods are highly comparable with a key difference in data analysis [17]: MMQPCR involves normalization against a reference gene, whereas standard qPCR relies on comparisons of the threshold cycle (Ct) values.

Infants younger than three months were excluded from our study for several reasons. First, telomere length at birth is highly variable, as reported in multiple studies [18,19,20,21]. This variability is consistent with observations of high telomere length heterogeneity in human embryos [22] and fetuses [23,24]. Moreover, neonatal telomere length has been shown to correlate with parental telomere length, although it remains controversial whether maternal [25,26] or paternal inheritance [27,28] exerts greater influence. Additionally, a longitudinal study in an SMA mouse model demonstrated that neurodevelopmental delay begins in late fetal life and neonatal period [29]. Considering these factors, we excluded early infancy from our cohort. The final mean age of our cases was approximately 60 months, with 53.4% female and 46.6% male participants. Given that the *SMN1* gene is located on autosomal chromosome 5 and the data of the SMA incidence were nearly equal between males and females [30], the sex distribution of our patients was in line with expectations.

In the Kashmir study, telomere length was found to be significantly shorter in SMA patients compared with healthy controls [16]. In our analysis of 58 SMA patients and 58 age- and sex-matched controls, no statistically significant difference in telomere length was observed. Notably, 32.8% (n = 19) of our patients had received gene replacement therapy with Onasemnogene abeparvovec-xioi (*ZOLGENSMA*). We hypothesized that the *SMN1* gene replacement could influence telomere length. Therefore, we reanalyzed our patient group by stratifying them according to treatment status. When the 39 untreated patients were compared with their matched controls, their telomeres were significantly shorter. In contrast, telomere length of the 19 patients treated with Onasemnogene abeparvovec did not differ significantly from controls’. Moreover, comparison between treated and untreated patients revealed a significant difference in telomere length, indicating the impact of the SMN protein deficiency on telomere length and the possible restorative effect of the replacement therapy. The existing literature has already demonstrated the benefits of the *SMN1* gene replacement therapy in survival, neuromotor development, and quality of life [31,32,33]. The findings of our research suggest that its favorable outcomes may also extend to telomere length.

The precise mechanism through which telomere attrition contributes to SMA pathogenesis remains unclear. Complete loss of the *SMN1* gene in SMA patients makes them fully dependent on the *SMN2* gene, a paralog that can exist in variable copy numbers, to produce the SMN protein necessary for the biogenesis and function of Cajal bodies [34]. Given that the assembly and maturation of the telomerase holoenzyme also occur in Cajal bodies [12,35], SMN deficiency could disrupt telomerase biogenesis by altering protein interactions in this compartment. A 2016 study demonstrated that SMN protein interacts with telomerase RNA and coilin within Cajal bodies and negatively regulates the binding of dyskerin to telomerase RNA, thereby modulating the assembly and function of the telomerase complex [13]. Consequently, the accumulation of short telomeres may fail to be compensated for by telomerase-mediated reverse transcription, leading to compromised telomere homeostasis. In addition, another study identified SMN as a telomerase-associated protein based on direct physical interactions confirmed by in vitro binding and immunoprecipitation assays [14]. These findings suggest that SMN deficiency in SMA may impair telomerase biogenesis or activity. Telomere dysfunction ultimately results in cellular senescence, cell cycle arrest, and apoptosis [36], which could contribute to increased morbidity and mortality in SMA.

Another mechanism by which telomere shortening may contribute to SMA pathogenesis is by promoting genomic instability and DNA damage, thereby exacerbating motor neuron degeneration. Several studies in the literature have explored the relationship between neurological disorders and telomere length [37]. With aging, senescent cells accumulate in the mouse brain, and this phenomenon is more pronounced in telomerase-deficient mice [38]. Leukocyte telomere length in patients with Alzheimer’s disease and dementia is shorter compared to age-matched healthy controls [39,40]. The relationship between telomere length and Parkinson’s disease remains inconclusive; however, telomeres shorter than 5 kilobases were reported to be found exclusively in the patient group [41]. In amyotrophic lateral sclerosis (ALS), a neurodegenerative disorder characterized by early mortality, reduced telomerase expression and shorter leukocyte telomeres were reported [42]. In addition, ALS patients with longer telomeres exhibited a 16% increase in median survival [43]. In transgenic ALS mouse models, increased telomerase expression was shown to delay disease onset and progression, whereas deletion of the telomerase reverse transcriptase gene resulted in earlier manifestation of the disease phenotype and reduced survival [44]. In a study conducted in 2024, telomere shortening has been shown to induce aging-related phenotypes in both motor neurons and astrocytes. These phenotypes include cellular senescence, increased inflammation, and DNA damage. In both developing and adult brain tissues, telomerase deficiency and telomere shortening have been reported to cause impaired neurogenesis, loss of neuronal differentiation, and increased susceptibility to neurodegenerative diseases [45].

Cellular senescence triggered by telomere shortening, and the resulting impairment in the regenerative capacity of motor neurons, has been proposed as another mechanism contributing to SMA pathogenesis [16]. The accumulation of senescent cells within a tissue not only limits growth and repair potential by reducing the number of mitotically active cells but also leads to the secretion of proteases, growth factors, and inflammatory cytokines that affect neighboring cells. This secretory activity, known as the senescence-associated secretory phenotype, is intended to promote immune-mediated clearance of senescent cells. However, with aging, the immune system’s ability to remove these cells declines [46].

Furthermore, mitochondrial dysfunction and oxidative stress develop during senescence, forming a positive feedback loop between reactive oxygen species and telomere damage. As dysfunctional mitochondria fail to meet cellular energy demands, cells lose their ability to maintain normal metabolic and physiological functions, eventually undergoing apoptosis or senescence [47,48]. In such a scenario, non-regenerative motor neurons may further exacerbate clinical deterioration in neurodegenerative diseases.

In the study by Hassan et al., SMA types were compared in terms of telomere length. The study concluded that telomere shortening was more pronounced in severe SMA forms (type 0, 1, and 2) compared to milder types such as type 3 and type 4 with no significant shortening observed. Thus, both the presence of telomere shortening in SMA and its association with disease severity were observed. Moreover, the authors suggested that telomere length could serve as a biomarker related to SMA severity and prognosis [16]. We also performed an analysis based on SMA types. The most common subtype in our cohort was SMA type 1, accounting for 63.8% (n = 37) of patients, consistent with the literature data of more than half of SMA patients having type 1 [30]. No significant telomere length differences were observed across SMA types. This may be attributable to differences in cohort sizes and ages across the groups. However, telomere lengths were significantly different between those who had received gene replacement therapy and those who had not in SMA type 1 patients. Additionally, type 2 patients without gene replacement therapy had significantly shorter telomeres, suggesting that SMA shortens telomeres, and the *SMN1* gene replacement potentially reverses this effect. Telomeres of patients with SMA type 3 did not differ significantly from their controls. The observation of prominent telomere shortening in severe SMA forms is consistent with the mechanism in which critically shortened telomeres may enhance motor neuron degeneration through DNA damage pathways.

Under normal circumstances, only about 10–15% of functional SMN protein is produced by *SMN2*; however, in SMA patients, the *SMN2* gene becomes the sole source of this crucial protein [1]. To our knowledge, this is the first study to investigate the relationship between the *SMN2* gene copy number and telomere length. No significant difference according to *SMN2* copy number was obtained in untreated cases; however, telomeres were significantly shorter compared with controls in patients with two *SMN2* copies. In contrast, those who had received gene therapy exhibited telomere lengths comparable to controls. Furthermore, a significant difference was observed when comparing telomere length between treated and untreated patients. These findings were interpreted as being consistent with our hypothesis. For patients carrying three or four *SMN2* copies, telomere length did not differ significantly from controls. This may be explained by the greater amount of SMN protein produced from higher copies of *SMN2*, which could contribute to the preservation of telomere length. However, since no studies in the literature have examined this relationship, further discussions cannot be had.

Ye et al. published the most comprehensive investigation into the relationship between telomere length and chronological age evaluating 414 studies encompassing 743,019 individuals. This analysis demonstrated that telomere length decreases with age, but this decline is not linear. Telomere shortening is most pronounced during childhood and early adulthood, with the rate of decline slowing after the age of 50 [49]. In this study, no significant correlation was detected between telomere length and age in controls. However, a weak but statistically significant negative correlation was observed in the patient group, indicating that telomeres shortened with increasing age. The demonstration of an age–telomere shortening correlation in a relatively small population suggests that SMA is a disease that significantly impacts telomere biology. According to SMA type, a moderate negative correlation was found. This may be explained by the higher mean age of patients with more advanced SMA types. No statistically significant correlation was found between *SMN2* copy number and telomere length. These findings suggest a slight age-related decline in telomere length among patients, but the effect was not pronounced and did not vary significantly with treatment status. To our knowledge, no prior studies have been published to compare with these findings.

Understanding how telomeres are affected in SMA may provide important insights into disease pathogenesis and therapeutic approaches. By including SMA patients treated with gene therapy in our study, we broadened its scope. We demonstrated that patients in whom the *SMN1* gene had been replaced exhibited telomere lengths similar to those of healthy controls. This finding highlights both the success of exogenous gene addition therapy using an AAV expressing *SMN1*, and the importance of SMN protein in maintaining telomere length. On the other hand, onasemnogene abeparvovec therapy presents several challenges [50]. These include the uncertainty regarding the duration of expression from the AAV transgene [51] and the dilution of AAV in dividing cells [52], which may reduce efficacy over time. Evaluating telomere length in patients who have received gene transfer therapy may serve as a predictive marker for expression decline and dilution, and could help identify patients who may require retreatment during long-term follow-up. A better understanding of the role of telomere shortening in SMA pathogenesis may also contribute to the development of novel therapeutic strategies aimed at preserving telomere length. Treatments designed to enhance telomerase activity may slow motor neuron degeneration and positively impact the course of the disease.

### Strengths and Limitations

A key strength of this study is the inclusion of a relatively large pediatric SMA cohort together with carefully age- and sex-matched healthy controls, which minimizes potential confounding and enhances the interpretability of the findings. Subgroup analyses were performed to explore associations with SMA type, the *SMN2* gene copy number, and patients’ gene replacement therapy status. Nevertheless, several limitations should be acknowledged. First, the cross-sectional design precludes causal inference and does not allow assessment of longitudinal telomere dynamics. Additionally, potential confounders such as nutritional status, environmental stressors, and comorbidities were not systematically controlled for. Second, telomere length was measured only in peripheral blood leukocytes, which may not fully reflect telomere biology in motor neurons or muscle tissue. Another limitation is that the AAV9 vector used for gene replacement therapy demonstrates variable transduction efficiency across tissues, with limited tropism for hematopoietic cells. Therefore, the leukocyte population analyzed by qPCR may represent a mixture of transduced and non-transduced cells, potentially underestimating telomere effects related to gene therapy. Finally, although the overall cohort size was relatively large, it remained limited for certain subgroup comparisons.

## 4. Materials and Methods

The study was approved by the Non-Interventional Clinical Research Ethics Committee of Necmettin Erbakan University (protocol code 2023/4202, date of approval: 17 February 2023). This study is based on the corresponding author’s Medical Genetics residency thesis and has not appeared in any journal publication [53]. Written informed consent was obtained from the legal guardians of all pediatric participants, and assent was obtained from children aged 8 years and older.

### 4.1. Sample Collection and Study Population

The case group in this study comprised individuals diagnosed with Spinal Muscular Atrophy (SMA), who were recruited from outpatient clinics of three centers: The Department of Medical Genetics, Necmettin Erbakan University Medical Faculty Hospital; the Division of Pediatric Neurology, Eskişehir Osmangazi University Medical Faculty Hospital; and the Division of Pediatric Pulmonology, Istanbul Medipol University Medipol Mega Hospital. Eligible participants with SMA were between 3 months and 18 years of age at the time of blood collection, had a genetically confirmed diagnosis, and no comorbidities unrelated to SMA. A total of 58 SMA patients met the inclusion criteria. Patients’ characteristics collected include age, sex, SMA clinical type, gene replacement treatment status, any diseases besides SMA, and genetic information (*SMN1* and *SMN2* copy numbers, determined using multiplex ligation-dependent probe amplification as part of the routine diagnostic workup). Nineteen of them had received gene replacement therapy before the age of 2, while the others were receiving standard-of-care treatments such as nusinersen or risdiplam. The control group consisted of 58 children who presented to Necmettin Erbakan University Medical Faculty Hospital. They had no major genetic or chronic disorders and were closely matched to the patients in terms of age (within ±3 months), sex, and ethnicity.

### 4.2. Genomic DNA Isolation

Peripheral blood samples (~2 mL) were collected from all participants into EDTA (ethylene diamine tetraacetic acid) tubes. Genomic DNA was isolated using the DiaRex Whole Blood Genomic DNA Extraction Kit II (Diagen Biotechnology, Ankara, Turkey) following the manufacturer’s protocol. DNA purity was evaluated using a NanoDrop spectrophotometer (Thermo Fisher Scientific, Waltham, MA, USA).

### 4.3. Quantification of Relative Telomere Length

Relative telomere length was determined by quantitative real-time PCR (qPCR) using a CFX96 Touch Real-Time PCR Detection System (Bio-Rad, Hercules, CA, USA). Telomeric sequences and a single-copy reference gene (*IFNB1*) were amplified using specific primers (Table 5). Each 20 µL reaction contained SensiFAST SYBR Master Mix—No ROX (Meridian Bioscience Inc., Cincinnati, OH, USA), primer mix, and genomic DNA. The thermal cycling profile consisted of an initial denaturation at 95 °C for 10 min, followed by 40 cycles of denaturation at 95 °C for 20 s, annealing at 52 °C for 20 s, and extension at 72 °C for 30 s. Amplification specificity was confirmed by melting curve analysis performed at 95 °C for 10 s, followed by a gradual temperature increase from 65 °C to 95 °C in 0.5 °C increments every 5 s.

Following amplification, Ct values were obtained for both the telomere and reference gene *IFNB1*. The Ct value represents the number of cycles required for the fluorescence signal to cross a predefined threshold. First, the difference between the Ct values of the target and reference genes was calculated (ΔCt = Ct_telomere − Ct_IFNB1). Then, the ΔCt of the experimental group was compared to the ΔCt of the control group to obtain ΔΔCt (ΔΔCt = ΔCt_SMA − ΔCt_control). The fold change in telomere length was finally calculated using the formula 2^−ΔΔCt^.

### 4.4. Statistical Analysis

Relative telomere length data were analyzed using IBM SPSS Statistics version 29.0.2.0 (IBM Corp., Armonk, NY, USA). Each DNA sample was analyzed in at least three technical replicates. The normality of data distribution was evaluated using the Kolmogorov–Smirnov and Shapiro–Wilk tests. Continuous variables were summarized as mean ± standard deviation for normally distributed data and as median (minimum–maximum) for non-normally distributed data. Comparisons between two independent groups were performed using Student’s *t*-test for normally distributed variables or the Mann–Whitney *U* test for non-normally distributed variables. For comparisons among more than two groups, the Kruskal–Wallis *H* test followed by Bonferroni post hoc correction was applied. Correlations between relative telomere length and continuous clinical variables were assessed using Spearman’s rank correlation coefficient. A *p* value < 0.05 was considered statistically significant.

## 5. Conclusions

In summary, pediatric patients with SMA exhibited significantly shorter telomeres than healthy controls, and gene replacement therapy may have potential restorative effects on telomere maintenance. These findings contribute to the emerging evidence linking telomere biology with neuromuscular and neurodegenerative disorders. Future longitudinal studies are warranted to clarify whether telomere length has clinical utility as a potential biomarker for disease severity or therapeutic response in SMA.

## Figures and Tables

**Table 1:** Mean ages of the study participants, according to gene replacement therapy status, SMA types and the *SMN2* gene copy numbers.

Participants (n, %)	Mean Age (Months) ± SD	Median Age (Months)	Minimum (Months)	Maximum (Months)
Patient Group (n = 58)	-	44.9	7.5	208.3
Untreated (n = 39)	-	53.1	7.5	208.3
Treated (n = 19)	42.1 ± 19.1	-	15.3	74.7
SMA type 1 (n = 37, 63.8%)	-	34.2	7.5	114.1
Treated (n = 18, 48.6%)	42.8 ± 18.4	-	-	-
Untreated (n = 19, 51.4%)	-	33.6	7.5	114.1
SMA type 2 (n = 11, 19%)	75.3 ± 52.9	-	-	-
Treated (n = 1, 9.1%)	30.2	-	-	-
Untreated (n = 10, 90.9%)	79.9 ± 53.5	-	-	-
SMA type 3 (n = 9, 15.5%)	113.7 ± 72.8	-	-	-
SMA type 4 (n = 1, 1.7%)	20.4	-	-	-
Two *SMN2* copies (n = 41, 70.7%)	-	38.6	7.5	183.2
Treated (n = 18, 43.9%)	42.8 ± 19.5	-	-	-
Untreated (n = 23, 56.1%)	-	34.2	7.5	183.2
Three *SMN2* copies (n = 13, 22.4%)	-	65.6	26.9	185
Treated (n = 1, 7.7%)	30.2	-	-	-
Untreated (n = 12, 92.3%)	72.8 ± 44.5	-	-	-
Four *SMN2* copies (n = 4, 6.9%)	146.5 ± 86.9	-	-	-
Control Group (n = 58)	-	43.5	8	212.5
TOTAL	-	44.9	7.5	212.5

SD: Standard Deviation.

**Table 2:** Comparison results of telomere length across specified groups according to gene replacement therapy status.

Group	Comparison Group	*p* Value
Patient group (n = 58)	Control group	0.346 *
Untreated patients (n = 39)	Their matched controls	0.029 *
Treated patients (n = 19)	Their matched controls	0.108 *
Treated patients (n = 19)	Untreated patients (n = 39)	**0.012 ^+^**

* Mann–Whitney *U* test and ^+^ Student’s *t* test were used for comparison between groups. Statistically significant results (*p* value < 0.05) are shown in bold.

**Table 3:** Evaluation of telomere length by SMA type across various comparison groups according to gene replacement therapy status.

Group	Comparison Group	*p* Value
SMA type 1 patients (n = 37)	Matched controls	0.846 *
Untreated (n = 19)	Matched controls	0.14 *
Treated (n = 18)	Matched controls	0.09 *
Treated (n = 18)	Untreated (n = 19)	**0.032 ^+^**
SMA type 2 patients (n = 11)	Matched controls	0.004 *
Untreated (n = 10)	Matched controls	0.002 *
SMA type 3 patients (n = 9)	Matched controls	0.757 *

* Mann–Whitney *U* test and ^+^ Student’s *t* test were used for comparison between groups. Statistically significant results (*p* value < 0.05) are shown in bold.

**Table 4:** Evaluation of telomere length by the SMN2 gene copy number across various comparison groups.

Group	Comparison Group	*p* Value *
Patients with two *SMN2* copies (n = 41)	Matched controls	0.613
Untreated (n = 23)	Matched controls	**0.024**
Treated (n = 18)	Matched controls	0.9
Treated (n = 18)	Untreated (n = 23)	**0.01**
Patients with three *SMN2* copies (n = 13)	Matched controls	0.521
Untreated (n = 12)	Matched controls	0.273
Patients with four *SMN2* copies (n = 4)	Matched controls	0.773

* Mann–Whitney *U* test was used for comparison between groups. Statistically significant results (*p* value < 0.05) are shown in bold.

**Table 5:** Primer sequences used in this study.

Primer Name	Nucleotide Sequence (5′ → 3′)
TEL-F	CGGTTTGTTTGGGTTTGGGTTTGGGTTTGGGTTTGGGTT
TEL-R	GGCTTGCCTTACCCTTACCCTTACCCTTACCCTTACCCT
IFNB1-F	TGGCACAACAGGTAGTAGGCGACAC
IFNB1-R	GCACAACAGGAGAGCAATTTGGAGGA

## Data Availability

The data presented in this study are available on request from the corresponding author due to privacy and ethical restrictions.

## Supplementary Materials

The following supporting information can be downloaded at: https://www.mdpi.com/article/10.3390/ijms262211223/s1.

## Abbreviations

The following abbreviations are used in this manuscript:

SMA	Spinal Muscular Atrophy
PCR	Polymerase Chain Reaction
SMN	Survival Motor Neuron
RNA	Ribonucleic Acid
mRNA	Messenger RNA
AAV	Adeno-Associated Viral Vector
snRNP	Small Nuclear Ribonucleoprotein
DNA	Deoxyribonucleic Acid
qPCR	Quantitative Polymerase Chain Reaction
MMQPCR	Monochrome Multiplex Quantitative Polymerase Chain Reaction
Ct	Threshold Cycle
ALS	Amyotrophic Lateral Sclerosis
EDTA	Ethylenediamine Tetraacetic Acid

## References

[1] Kolb S.J., Kissel J.T. (2015). Spinal Muscular Atrophy. Neurol. Clin..

[2] Monani U.R., Lorson C.L., Parsons D.W., Prior T.W., Androphy E.J., Burghes A.H., McPherson J.D. (1999). A single nucleotide difference that alters splicing patterns distinguishes the SMA gene SMN1 from the copy gene SMN2. Hum. Mol. Genet..

[3] Mercuri E., Sumner C.J., Muntoni F., Darras B.T., Finkel R.S. (2022). Spinal muscular atrophy. Nat. Rev. Dis. Primers.

[4] Finkel R.S., Mercuri E., Darras B.T., Connolly A.M., Kuntz N.L., Kirschner J., Chiriboga C.A., Saito K., Servais L., Tizzano E. (2017). Nusinersen versus Sham Control in Infantile-Onset Spinal Muscular Atrophy. N. Engl. J. Med..

[5] Ratni H., Ebeling M., Baird J., Bendels S., Bylund J., Chen K.S., Denk N., Feng Z., Green L., Guerard M. (2018). Discovery of Risdiplam, a Selective Survival of Motor Neuron-2 (SMN2) Gene Splicing Modifier for the Treatment of Spinal Muscular Atrophy (SMA). J. Med. Chem..

[6] Mendell J.R., Al-Zaidy S., Shell R., Arnold W.D., Rodino-Klapac L.R., Prior T.W., Lowes L., Alfano L., Berry K., Church K. (2017). Single-Dose Gene-Replacement Therapy for Spinal Muscular Atrophy. N. Engl. J. Med..

[7] Cooper K., Nalbant G., Sutton A., Harnan S., Thokala P., Chilcott J., McNeill A., Bessey A. (2024). Systematic Review of Presymptomatic Treatment for Spinal Muscular Atrophy. Int. J. Neonatal Screen..

[8] Turner K.J., Vasu V., Griffin D.K. (2019). Telomere Biology and Human Phenotype. Cells.

[9] Huang X., Huang L., Lu J., Cheng L., Wu D., Li L., Zhang S., Lai X., Xu L. (2025). The relationship between telomere length and aging-related diseases. Clin. Exp. Med..

[10] Greider C.W., Blackburn E.H. (1989). A telomeric sequence in the RNA of Tetrahymena telomerase required for telomere repeat synthesis. Nature.

[11] Shay J.W., Wright W.E. (2019). Telomeres and telomerase: Three decades of progress. Nat. Rev. Genet..

[12] Machyna M., Heyn P., Neugebauer K.M. (2013). Cajal bodies: Where form meets function. Wiley Interdiscip. Rev. RNA.

[13] Poole A.R., Hebert M.D. (2016). SMN and coilin negatively regulate dyskerin association with telomerase RNA. Biol. Open.

[14] Bachand F., Boisvert F.M., Cote J., Richard S., Autexier C. (2002). The product of the survival of motor neuron (SMN) gene is a human telomerase-associated protein. Mol. Biol. Cell.

[15] Lansdorp P.M. (2008). Telomeres, stem cells, and hematology. Blood.

[16] Hassan R., Bhat G.R., Mir F.A., Ganie H.A., Mushtaq I., Bhat M.A., Asimi R.P., Afroze D. (2024). Concomitant telomere attrition is associated with spinal muscular atrophy in highly inbred region of North India: Unraveling the thread in Kashmir region. BMC Med. Genom..

[17] Martin N.A., McLester-Davis L.W.Y., Roy T.R., Magruder M.G., Hastings W.J., Drury S.S. (2024). Monochrome Multiplex Quantitative PCR Telomere Length Measurement. J. Vis. Exp. JoVE.

[18] Friedrich U., Schwab M., Griese E.U., Fritz P., Klotz U. (2001). Telomeres in neonates: New insights in fetal hematopoiesis. Pediatr. Res..

[19] Menon R., Yu J., Basanta-Henry P., Brou L., Berga S.L., Fortunato S.J., Taylor R.N. (2012). Short fetal leukocyte telomere length and preterm prelabor rupture of the membranes. PLoS ONE.

[20] Okuda K., Bardeguez A., Gardner J.P., Rodriguez P., Ganesh V., Kimura M., Skurnick J., Awad G., Aviv A. (2002). Telomere length in the newborn. Pediatr. Res..

[21] Vasu V., Turner K.J., George S., Greenall J., Slijepcevic P., Griffin D.K. (2017). Preterm infants have significantly longer telomeres than their term born counterparts. PLoS ONE.

[22] Turner S., Wong H.P., Rai J., Hartshorne G.M. (2010). Telomere lengths in human oocytes, cleavage stage embryos and blastocysts. Mol. Hum. Reprod..

[23] Holmes D.K., Bellantuono I., Walkinshaw S.A., Alfirevic Z., Johnston T.A., Subhedar N.V., Chittick R., Swindell R., Wynn R.F. (2009). Telomere length dynamics differ in foetal and early post-natal human leukocytes in a longitudinal study. Biogerontology.

[24] Youngren K., Jeanclos E., Aviv H., Kimura M., Stock J., Hanna M., Skurnick J., Bardeguez A., Aviv A. (1998). Synchrony in telomere length of the human fetus. Hum. Genet..

[25] Akkad A., Hastings R., Konje J.C., Bell S.C., Thurston H., Williams B. (2006). Telomere length in small-for-gestational-age babies. BJOG Int. J. Obstet. Gynaecol..

[26] Factor-Litvak P., Susser E., Kezios K., McKeague I., Kark J.D., Hoffman M., Kimura M., Wapner R., Aviv A. (2016). Leukocyte Telomere Length in Newborns: Implications for the Role of Telomeres in Human Disease. Pediatrics.

[27] Njajou O.T., Cawthon R.M., Damcott C.M., Wu S.H., Ott S., Garant M.J., Blackburn E.H., Mitchell B.D., Shuldiner A.R., Hsueh W.C. (2007). Telomere length is paternally inherited and is associated with parental lifespan. Proc. Natl. Acad. Sci. USA.

[28] Nordfjäll K., Larefalk A., Lindgren P., Holmberg D., Roos G. (2005). Telomere length and heredity: Indications of paternal inheritance. Proc. Natl. Acad. Sci. USA.

[29] Kong L., Valdivia D.O., Simon C.M., Hassinan C.W., Delestrée N., Ramos D.M., Park J.H., Pilato C.M., Xu X., Crowder M. (2021). Impaired prenatal motor axon development necessitates early therapeutic intervention in severe SMA. Sci. Transl. Med..

[30] Verhaart I.E.C., Robertson A., Wilson I.J., Aartsma-Rus A., Cameron S., Jones C.C., Cook S.F., Lochmüller H. (2017). Prevalence, incidence and carrier frequency of 5q-linked spinal muscular atrophy—A literature review. Orphanet J. Rare Dis..

[31] Strauss K.A., Farrar M.A., Muntoni F., Saito K., Mendell J.R., Servais L., McMillan H.J., Finkel R.S., Swoboda K.J., Kwon J.M. (2022). Onasemnogene abeparvovec for presymptomatic infants with three copies of SMN2 at risk for spinal muscular atrophy: The Phase III SPR1NT trial. Nat. Med..

[32] Strauss K.A., Farrar M.A., Muntoni F., Saito K., Mendell J.R., Servais L., McMillan H.J., Finkel R.S., Swoboda K.J., Kwon J.M. (2022). Onasemnogene abeparvovec for presymptomatic infants with two copies of SMN2 at risk for spinal muscular atrophy type 1: The Phase III SPR1NT trial. Nat. Med..

[33] Alajjuri M.A., Abusamra R., Mundada V., Narayan O. (2024). Real-World Data in Children with Spinal Muscular Atrophy Type 1 on Long-Term Ventilation Receiving Gene Therapy: A Prospective Cohort Study. Adv. Respir. Med..

[34] Hebert M.D., Szymczyk P.W., Shpargel K.B., Matera A.G. (2001). Coilin forms the bridge between Cajal bodies and SMN, the spinal muscular atrophy protein. Genes Dev..

[35] Jády B.E., Bertrand E., Kiss T. (2004). Human telomerase RNA and box H/ACA scaRNAs share a common Cajal body-specific localization signal. J. Cell Biol..

[36] Di Micco R., Krizhanovsky V., Baker D., d’Adda di Fagagna F. (2021). Cellular senescence in ageing: From mechanisms to therapeutic opportunities. Nat. Rev. Mol. Cell Biol..

[37] Rossiello F., Jurk D., Passos J.F., d’Adda di Fagagna F. (2022). Telomere dysfunction in ageing and age-related diseases. Nat. Cell Biol..

[38] Jurk D., Wang C., Miwa S., Maddick M., Korolchuk V., Tsolou A., Gonos E.S., Thrasivoulou C., Saffrey M.J., Cameron K. (2012). Postmitotic neurons develop a p21-dependent senescence-like phenotype driven by a DNA damage response. Aging Cell.

[39] Grodstein F., van Oijen M., Irizarry M.C., Rosas H.D., Hyman B.T., Growdon J.H., De Vivo I. (2008). Shorter telomeres may mark early risk of dementia: Preliminary analysis of 62 participants from the nurses’ health study. PLoS ONE.

[40] Thomas P., O’Callaghan N.J., Fenech M. (2008). Telomere length in white blood cells, buccal cells and brain tissue and its variation with ageing and Alzheimer’s disease. Mech. Ageing Dev..

[41] Guan J.Z., Maeda T., Sugano M., Oyama J., Higuchi Y., Suzuki T., Makino N. (2008). A percentage analysis of the telomere length in Parkinson’s disease patients. J. Gerontol. Ser. A Biol. Sci. Med. Sci..

[42] De Felice B., Annunziata A., Fiorentino G., Manfellotto F., D’Alessandro R., Marino R., Borra M., Biffali E. (2014). Telomerase expression in amyotrophic lateral sclerosis (ALS) patients. J. Hum. Genet..

[43] Al Khleifat A., Iacoangeli A., Shatunov A., Fang T., Sproviero W., Jones A.R., Opie-Martin S., Morrison K.E., Shaw P.J., Shaw C.E. (2019). Telomere length is greater in ALS than in controls: A whole genome sequencing study. Amyotroph. Lateral Scler. Front. Degener..

[44] Eitan E., Tichon A., Gazit A., Gitler D., Slavin S., Priel E. (2012). Novel telomerase-increasing compound in mouse brain delays the onset of amyotrophic lateral sclerosis. EMBO Mol. Med..

[45] Harley J., Santosa M.M., Ng C.Y., Grinchuk O.V., Hor J.H., Liang Y., Lim V.J., Tee W.W., Ong D.S.T., Ng S.Y. (2024). Telomere shortening induces aging-associated phenotypes in hiPSC-derived neurons and astrocytes. Biogerontology.

[46] Muñoz-Espín D., Serrano M. (2014). Cellular senescence: From physiology to pathology. Nat. Rev. Mol. Cell Biol..

[47] Harrington J.S., Ryter S.W., Plataki M., Price D.R., Choi A.M.K. (2023). Mitochondria in health, disease, and aging. Physiol. Rev..

[48] Sahin E., Colla S., Liesa M., Moslehi J., Müller F.L., Guo M., Cooper M., Kotton D., Fabian A.J., Walkey C. (2011). Telomere dysfunction induces metabolic and mitochondrial compromise. Nature.

[49] Ye Q., Apsley A.T., Etzel L., Hastings W.J., Kozlosky J.T., Walker C., Wolf S.E., Shalev I. (2023). Telomere length and chronological age across the human lifespan: A systematic review and meta-analysis of 414 study samples including 743,019 individuals. Ageing Res. Rev..

[50] René C.A., Parks R.J. (2023). Expanding the Availability of Onasemnogene Abeparvovec to Older Patients: The Evolving Treatment Landscape for Spinal Muscular Atrophy. Pharmaceutics.

[51] Thomsen G., Burghes A.H.M., Hsieh C., Do J., Chu B.T.T., Perry S., Barkho B., Kaufmann P., Sproule D.M., Feltner D.E. (2021). Biodistribution of onasemnogene abeparvovec DNA, mRNA and SMN protein in human tissue. Nat. Med..

[52] Alves C.R.R., Zhang R., Johnstone A.J., Garner R., Eichelberger E.J., Lepez S., Yi V., Stevens V., Poxson R., Schwartz R. (2020). Whole blood survival motor neuron protein levels correlate with severity of denervation in spinal muscular atrophy. Muscle Nerve.

[53] Okur Altındaş B. (2025). Spinal Musküler Atrofi Hastalarında Telomer Uzunluğunun Değerlendirilmesi. Residency Thesis.

