# Supplementary material for: Evaluation of the Telomere Length in Patients with Spinal Muscular Atrophy
Source: Int J Mol Sci. 2025 Nov 20;26(22):11223. doi: 10.3390/ijms262211223 (PMC12653482; doi:10.3390/ijms262211223)
Supplement: Supplementary file 1 [file ijms-26-11223-s001.zip › ijms-3945338-supplementary.pdf]

Table S1: The fold change values of all study participants, representing the log_2-transformed relative telomere length obtained using the formula $2^{-\Delta\Delta Ct}$.

Patients	Fold Change Values	Controls	Fold Change Values
1	-2,45203125	1	-3,53203125
2	-3,97203125	2	-3,64203125
3	-3,16203125	3	4,75796875
4	-4,03203125	4	2,39796875
5	-2,89203125	5	-0,00203125
6	0,58796875	6	9,52796875
7	0,65796875	7	1,11796875
8	-3,35203125	8	2,83796875
9	1,10796875	9	1,91796875
10	0,97796875	10	2,39796875
11	-0,00203125	11	1,85796875
12	-1,46203125	12	-3,91203125
13	-3,39203125	13	-2,67203125
14	-1,85203125	14	-3,35203125
15	-2,33203125	15	-1,24203125
16	3,08796875	16	-2,43203125
17	5,96796875	17	4,25796875
18	-0,32203125	18	-6,55203125
19	0,69796875	19	-3,43203125
20*	3,08796875	20	1,25796875
21*	2,40796875	21	-3,38203125
22*	-1,44203125	22	-1,53203125
23*	-1,22203125	23	-0,24203125
24*	1,18796875	24	-2,28203125
25*	0,94796875	25	-3,82203125
26*	-2,15203125	26	-1,63203125

27	0,86796875	27	2,29796875
28*	2,04796875	28	15,64796875
29	0,39796875	29	-0,19203125
30*	5,32796875	30	0,57796875
31	0,58796875	31	0,74796875
32*	1,21796875	32	2,12796875
33*	0,08796875	33	-2,15203125
34*	0,80796875	34	0,13796875
35*	2,54796875	35	-6,87203125
36*	1,61796875	36	2,53796875
37*	-2,67203125	37	-2,32203125
38*	-2,76203125	38	-0,39203125
39*	3,85796875	39	-0,86203125
40	-0,75203125	40	2,44796875
41	1,58796875	41	2,30796875
42*	1,23796875	42	2,07796875
43	1,46796875	43	-3,01203125
44	-1,30203125	44	0,48796875
45*	0,74796875	45	1,58796875
46	-8,17203125	46	0,84796875
47	2,74796875	47	3,58796875
48	1,76796875	48	3,30796875
49	-0,79203125	49	-5,87203125
50	0,38796875	50	0,85796875
51	0,44796875	51	3,05796875
52	-1,10203125	52	1,21796875
53	-4,60203125	53	2,83796875
54	-0,18203125	54	-1,81203125
55	-3,56203125	55	1,01796875

56	-2,31203125	56	-8,57203125
57	-2,17203125	57	3,55796875
58	-5,47203125	58	2,52796875

* indicates patients who received gene replacement therapy.
